# Modulation of Autophagy-Like Processes by Tumor Viruses

**DOI:** 10.3390/cells1030204

**Published:** 2012-06-25

**Authors:** Hildegard I. D. Mack, Karl Munger

**Affiliations:** Division of Infectious Diseases, Brigham and Women’s Hospital, Department of Medicine, Harvard Medical School, Boston, MA 02115, USA

**Keywords:** autophagy, cancer, Epstein–Barr virus, hepatitis B virus, hepatitis C virus, human papillomavirus, human T-lymphotropic virus 1, Kaposi’s sarcoma-associated herpesvirus, Merkel cell polyomavirus

## Abstract

Autophagy is an intracellular degradation pathway for long-lived proteins and organelles. This process is activated above basal levels upon cell intrinsic or environmental stress and dysregulation of autophagy has been linked to various human diseases, including those caused by viral infection. Many viruses have evolved strategies to directly interfere with autophagy, presumably to facilitate their replication or to escape immune detection. However, in some cases, modulation of autophagy appears to be a consequence of the virus disturbing the cell’s metabolic signaling networks. Here, we summarize recent advances in research at the interface of autophagy and viral infection, paying special attention to strategies that human tumor viruses have evolved.

## 1. Introduction

### 1.1. Human Oncogenic Viruses

Viruses are intracellular parasites that strictly depend on a host to replicate. Thus, after entry, they reprogram their host cells to meet their needs. For the host, the consequences of viral infection span a wide range from being asymptomatic to causing deadly disease. Approximately 10–15% of human cancers are associated with viral infections [1]. Despite this substantial number, the list of human viruses that are clearly involved in the etiology of human tumors is rather short [2]. The first human tumor viruses to be identified is Epstein–Barr Virus (EBV) that has been linked to Hodgkin’s lymphoma, Burkitt’s lymphoma, nasopharyngeal carcinoma as well as other hematological malignancies in immunosuppressed individuals [3]. Hepatitis B (HBV) and C (HCV) viruses have been linked to hepatocellular carcinoma (HCC) [4], human papillomaviruses (HPVs) to cervical, anal, vulvar, vaginal, penile and oropharyngeal cancers as well as to squamous cell skin carcinomas in immunosuppressed patients [5], and the human T-lymphotropic virus 1 (HTLV-1) has been linked to adult T-cell leukemia (ATL) [6]. More recently identified human oncogenic viruses include Kaposi’s sarcoma-associated herpesvirus (KSHV) that causes Kaposi’s sarcoma, primary effusion lymphoma (PEL) and Castleman’s disease [7] and the Merkel cell polyomavirus (MCPyV) that has been linked to a rare but highly lethal skin cancer, Merkel cell carcinoma (MCC) [2] (Table 1). In addition to the viruses listed above, a number of other viruses have been reported to contribute to human cancers but these associations remain to be confirmed [8]. One interesting emerging candidate is the human cytomegalovirus (HCMV), which may modulate the carcinogenic phenotype of cancers, in particular glioblastoma. While HCMV encodes multiple proteins that can subvert the activities of cellular tumor suppressors, the clinical significance of the presence of HCMV sequences in these tumors remains controversial [9]. With the advent of deep sequencing, one might expect that viral sequences and/or transcripts will be detected in even more cancer types but the mere discovery of viral sequences in a tumor does not imply a causative relationship. It is worth noting that while infections with one of the established human tumor viruses is associated with a majority or at least a significant percentage of the respective cancers, some of these cancers can also arise without viral infection. 

Although human tumor viruses comprise a diverse group of viruses (Table 1), most of them share the ability to establish long-term latent or persistent infections. In this state, the viral genome is maintained as an episomal element or as an integrated genome copy within a host chromosome, and is replicated along with the host cellular genome by the host’s DNA replication machinery. Structural viral proteins required for virion formation are not, or at least not abundantly synthesized during latency [2]. This most likely decreases the vulnerability of virally infected cells for elimination by the immune system. 

To ensure viral survival and propagation, progeny virus needs to be generated and the host cell has to produce the necessary enzymes and metabolites. Therefore, many tumor viruses encode proteins that promote cell cycle entry, counteract programmed cell death, subvert cellular differentiation and/or interfere with immune signaling [10]. However, virtually all of the cellular pathways targeted by these viruses can contribute to carcinogenesis. In some cases, particularly with HPV associated cervical carcinoma and MCPyV induced Merkel cell carcinoma, viral oncogenesis represents a consequence of a non-productive infection, *i.e.*, the expression of viral proteins in the absence of the viral life cycle and many of these tumors only express a subset of viral proteins or fragments thereof from integrated viral subgenomes [11].

**Table 1:** Human tumor viruses, their associated cancers, and mechanisms of autophagy modulation.

Associated cancer types	Mechanism of interference with autophagy and/or autophagy-regulating pathways
**Epstein–Barr virus (EBV)** *Herpesviridae*	
Burkitt’s and Hodgkin- and non-Hodgkin lymphomas, nasopharyngeal carcinoma, lymphoproliferative diseases	BILF1 ➔ PKR inhibition ➔ may inhibit autophagy [12]
	LMP1 ➔ JNK activation ➔ may promote autophagy [13,14,15]
	LMP1 ➔ NF-κB activation ➔ inhibits autophagy in B cells [16]
	LMP1 ➔ p38 activation ➔ may inhibit autophagy [17]
	LMP1 ➔ activation of UPR signaling ➔ autophagic markers [18,19]
	LMP1/LMP2A ➔ PI3K/mTOR activation ➔ may inhibit autophagy [20,21,22,23,24]
	ZTA ➔ NF-κB inhibition ➔ ? [25]
**Hepatitis B virus (HBV)** *Hepadnaviridae*	
Hepatocellular carcinoma	HBx ➔ increased Beclin-1 transcription ➔ autophagic markers [26]
	HBx ➔ increased LC3-lipidation and VPS34 activity ➔ incomplete autophagic response [27]
	HBx ➔ interacts with p53 ➔ may inhibit autophagy [28]
	HBx ➔ p38 activation ➔ may inhibit autophagy [29]
	HBx/LHBs/MHBs ➔ ERK activation ➔ may inhibit autophagy [30,31,32]
	LHBs ➔ PI3K/AKT/mTOR activation ➔ may inhibit autophagy [32,33,34]
	LHBs/MHBs ➔ NF-κB activation ➔ ? [31,35]
	SHBs ➔ activation of UPR signaling ➔ incomplete autophagic response [33,36]
	SHBs ➔ interacts with LC3 ➔ incomplete autophagic response [36]
**Hepatitis C virus (HCV)** *Flaviviridae*	
Hepatocellular carcinoma	Core/NS3 ➔ ERK activation ➔ may inhibit autophagy [37,38]
	Core/NS3 ➔ JNK activation ➔ may promote autophagy [37,38]
	Core/NS3/NS4B/NS5A ➔ NF-κB activation ➔ ? [37,39,40]
	Core/NS3 ➔ p38 activation ➔ may inhibit autophagy [37,38]
	Core/NS3/NS5A ➔ interact with p53 ➔ may inhibit autophagy [41,42,43,44,45]
	Core/NS4B ➔ AKT activation ➔ may inhibit autophagy [46]
	NS3 ➔ interacts with IRGM ➔ increases autophagic markers [47]
	NS4B ➔ activation of UPR signaling ➔ autophagic markers [48]
	NS4B ➔ interacts with Rab5 and VPS34 ➔ incomplete autophagic response [48]
	NS5A ➔ PI3K/mTOR activation ➔ may inhibit autophagy [49,50]
	NS5A ➔ ERK inhibition ➔ may activate autophagy [50,51]
	NS5A ➔ PKR inhibition ➔ may inhibit autophagy [52]
	NS5A ➔ p38 inhibition ➔ may promote autophagy [53]
	NS5B ➔ interacts with ATG5 ➔ ? [54]
	? ➔ increased Beclin-1 expression ➔ autophagic markers [55]
**Human papillomavirus, high-risk types (HPV)** *Papillomaviridae*	
Cervical, anal and penile cancers, head and neck cancers	E5 ➔ p38 activation ➔ may inhibit autophagy [56]
	E5/E6/E7 ➔ inhibit XBP1-splicing under basal conditions ➔ ? [57]
	E6 ➔ sustained AKT/mTORC1 activity ➔ may inhibit autophagy [58,59]
	E6 ➔ inhibits p53 ➔ may inhibit autophagy [60]
	E6/E7 ➔ ERK activation ➔ may inhibit autophagy [61]
	E7 ➔ AKT activation ➔ may inhibit autophagy [62,63]
	E7 ➔ NF-κB inhibition ➔ ? [64,65,66]
	E7 ➔ JNK inhibition ➔ may inhibit autophagy [67]
	E7 ➔ ? ➔ autophagic markers [68]
**Human T-lymphotropic virus (HTLV-1)** *Retroviridae*	
Adult T-cell leukemia	Tax ➔ JNK activation ➔ may promote autophagy [69]
	Tax ➔ sustained AKT/mTORC1 activity ➔ may inhibit autophagy [70,71]
	Tax ➔ p38 activation ➔ may inhibit autophagy [69]
	Tax ➔ activation of UPR signaling ➔ may promote autophagy [72]
	Tax ➔ inhibits p53 ➔ may inhibit autophagy [73]
	Tax+HBZ ➔ NF-κB activation ➔ ? [74,75,76,77]
**Kaposi’s sarcoma associated herpesvirus (KSHV)** *Herpesviridae*	
Kaposi’s sarcoma, pleural effusion lymphoma, multicentric Castleman’s disease	K1 ➔ PI3K/AKT/mTOR activation ➔ may inhibit autophagy [78,79,80]
	K15 ➔ ERK activation ➔ may inhibit autophagy [81]
	K15 ➔ p38 activation ➔ may inhibit autophagy [81]
	LANA ➔ inhibits p53 ➔ may inhibit autophagy [82]
	ORF45 ➔ sustained ERK/RSK activity ➔ autophagy [83,84]
	ORF49 ➔ JNK activation➔ may promote autophagy [85]
	ORF49/vGPCR ➔ p38 activation➔ may inhibit autophagy [85,86]
	RTA ➔? ➔ increased autophagy [87]
	vBCL-2 ➔ interacts with Beclin-1 ➔ autophagy inhibition [88,89]
	vFLIP ➔ interacts with ATG3 ➔ autophagy inhibition [90]
	vFLIP/K15/ORF75/miR-K1/vGPCR7/vIRF3 ➔ NF-κB activation ➔ ? [81,91,92,93,94,95,96]
	vGPCR ➔ PI3Kγ/mTORC1 activation ➔ may inhibit autophagy [97]
	vIRF2/vIRF3 ➔ PKR inhibition ➔ may inhibit autophagy [98,99]
	vIRF3 ➔ NF-κB inhibition ➔ ? [93,100]
**Merkel cell polyomavirus (MCPyV)** *Polyomaviridae*	
Merkel cell carcinoma	Small T ➔ mTORC1 activation ➔ may inhibit autophagy [75]

The table lists established human tumor viruses, their associated malignancies and the autophagy-related proteins and autophagy-regulating signaling pathways they modulate. Note that in many studies, an effect on autophagy has not been explicitly investigated or conclusively and comprehensively validated [101]. With regard to the current literature, it is difficult to predict effects of NF-kB activation on autophagy. ERK, JNK and p38 modulate or have been suggested to modulate autophagy via pathways other than NF-kB, and the predictions listed in this table are based on these mechanisms [102,103,104].

### 1.2. Autophagy—Basic Function and Role in Human Disease

Autophagy is a key homeostatic process conserved across all eukaryotic cells. The term literally translated means “self-eating” and describes a degradation pathway for intracellular structures via the lysosomal compartment. Although several mechanistically distinct forms of autophagy are distinguished (macro-, micro- and chaperone-mediated autophagy (reviewed in [105]) “autophagy” is commonly (and also for the purpose of this review) used synonymously with macroautophagy, the most widely studied subtype. (Macro)autophagy is characterized by formation of large, double membraned vesicles, so called autophagosomes, that sequester bulk portions of cytoplasm and, after closure, fuse with lysosomes so that the cargo can be degraded by lysosomal enzymes. This process takes place at a low basal level under physiologic conditions and facilitates turnover of long-lived proteins and organelles. However, upon exposure to environmental and endogenous stressors, such as nutrient- and growth factor deprivation, hypoxia, high temperature or organelle damage, autophagy is upregulated. Autophagy primarily represents a pro-survival mechanism, but in complex multicellular organisms, it serves additional purposes, including a role in adaptive and innate immunity. Autophagy has also been linked to programmed cell death, however, it is controversial at present whether the phenomenon of autophagic cell death actually exists [106]. Deregulation of autophagy has been implicated in a variety of human diseases including neurodegenerative diseases, cardiac disease, liver disease, myopathies, cancer and bacterial and viral infection [105]. Human tumors associated with the viruses mentioned above represent the interface between cancer and infectious diseases. Thus, autophagy may play an especially multifaceted role in virus-associated malignancies since it has pro- and anti-tumorigenic as well as pro- and anti-viral functions. 

### 1.3. Components of the Mammalian Autophagy Machinery

**Figure 1:**
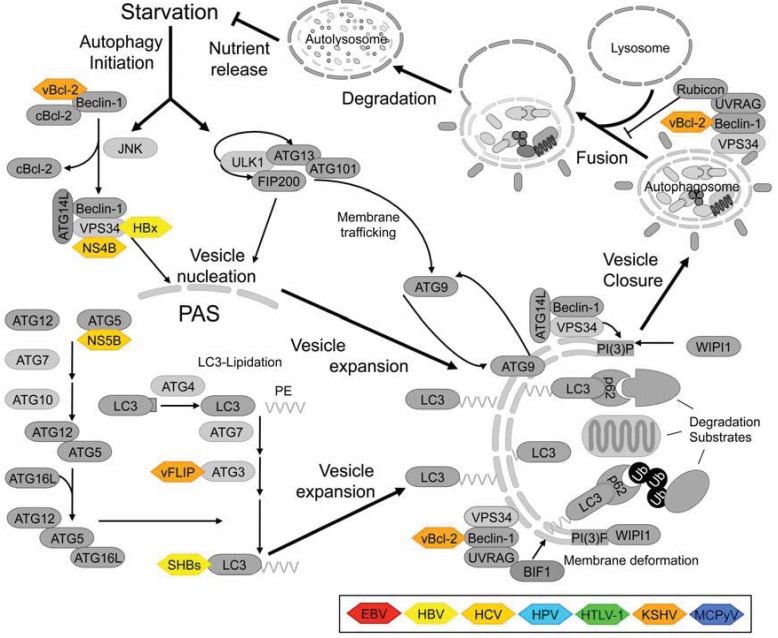
Schematic representation of the autophagy pathway in mammalian cells and interactions with viral proteins.

On the molecular level, autophagy is mediated by the so-called Atg-genes/proteins, most of which were originally identified in genetic screens in the yeast Saccharomyces cerevisiae [107]. Of the 35 Atg-proteins described to date, 21 function in all autophagy-related processes or specifically in non‑selective bulk macroautophagy, while 14 Atg-proteins are required only for specialized subtypes such as pexophagy or mitophagy or the yeast-specific cytoplasm to vacuole targeting (Cvt) pathway [108,109,110,111,112]. Mammalian cells possess structural or functional homologs for at least 16 of the 21 “core” Atg-proteins and a few vertebrate- or mammalian-specific autophagy factors such as Ambra-1 or ATG101 [108,113,114,115]. An overview of the autophagy pathway in mammalian cells is presented in Figure 1. 

In this review, we summarize our current knowledge on the particular strategies that human tumor viruses have evolved to interface with the host cell autophagy machinery and with autophagy-regulating signaling pathways. We first examine the role of autophagy on the viral life cycle and subsequently describe mechanisms by which tumor viruses modulate this process. Yet, it should be emphasized that most of the virus—host cell interactions discussed below are not specific to human tumor viruses but have been described for non-tumorigenic viruses as well. 

The Atg-proteins can be grouped into four (or into six, according to other authors, e.g., [116]) functional modules that mediate the major steps of autophagy [117]: (1) An autophagy initiation complex consisting of the Serine/Threonine kinase ULK1 (or its close relative ULK2), and the regulatory proteins ATG13, focal adhesion kinase family interacting protein of 200 kD (FIP200) and ATG101 [116]; (2) A class III phosphatidylinositol 3-kinase (PI3K), VPS34, which, in cooperation with its interaction partners, facilitates vesicle nucleation. There are at least three biochemically and functionally distinct class III PI3K-complexes in mammalian cells, which share a common core comprising VPS34 itself and ATG6/Beclin-1 as well as p150 and Ambra-1 (omitted for clarity). The first complex comprises the core and ATG14L/Barkor and mediates autophagosome nucleation. The second complex, containing the core and UV irradiation resistance associated protein (UVRAG) may be involved in autophagosome extension and/or other endosomal pathways [118]. The third complex, in which the core is associated with UVRAG and RUN domain and cysteine-rich domain containing Beclin-1 interacting protein (Rubicon) inhibits autophagosome and endosome maturation; (3) Two interconnected ubiquitin-like conjugation systems involved in autophagosome expansion and cargo recruitment. In these systems, the E1- and E2-like enzymes, ATG7 and ATG10, respectively, facilitate conjugation of the ubiquitin-like protein ATG12 to ATG5. This ATG5-ATG12 conjugate is localized to the expanding autophagosomal membrane by ATG16L and potentially serves as the E3-ligase in the second conjugation system, which facilitates lipidation of the peripheral membrane protein LC3. Conjugation of LC3 further requires proteolytic processing by ATG4, as well as the E1-like enzyme ATG7 and another E2-like enzyme, ATG3 [117]. (4) A membrane trafficking and recycling system whose components are still poorly characterized functionally and include WD repeat domain phosphoinositide-interacting protein 1 (WIPI1), which binds phosphatidylinositol-3-phosphate (the product of the VPS34-lipid kinase reaction) and the putative membrane carrier protein ATG9 as well as another transmembrane protein, VMP1, and ATG2 (omitted for clarity) [117]. Some substrates are marked for autophagic degradation by ubiquitination, and both ubiquitinated and non-ubiquitinated cargo can be recruited to the autophagosome by LC3‑interacting adaptor proteins such as p62/sequestosome 1 [119]. Autophagic adaptors are especially important in selective forms autophagy and may also play a role in xenophagy of viral proteins [120]. Viral proteins that have been reported to physically interact with components of the autophagy machinery are depicted in this schematic drawing. Please note that upstream signaling pathways that target the ULK1-autophagy initiation complex or one of the other Atg-proteins and viral proteins interfering with them were omitted from this figure for clarity but are shown in detail in Figures 3-5. Some of the interactions of viral proteins with host cell autophagy-factors may require further experimental validation (see main text). Light grey rounded rectangles-host cell proteins with enzymatic activity; dark grey rounded rectangles-other host cell proteins; colored hexagons-viral proteins; : activation; to ⊥: inhibition.

## 2. Benefits of Autophagy Modulation on the Viral Life Cycle

**Figure 2:**
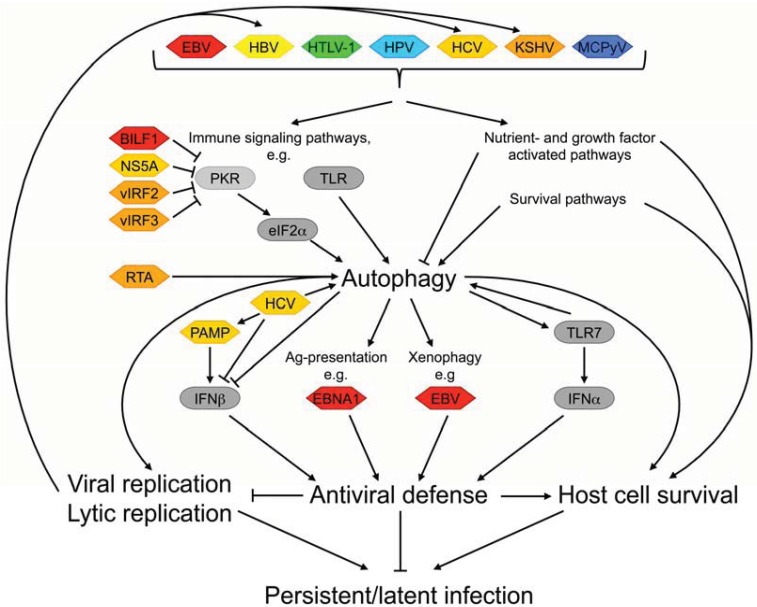
Autophagy and infection by human tumor viruses.

As with viral reprogramming of the host cell in general, viral interference with autophagy primarily aims to promote the viral life cycle. This includes avoiding detection by the immune system and creating a cellular environment favorable for viral replication. Autophagy is increasingly recognized as an important part of both innate and adaptive immune responses, which pathogens have to escape in order to successfully establish and sustain an infection [121]. This clearly provides a rationale for viruses to block autophagy. Yet, with regard to viral replication, the autophagy machinery appears to be beneficial rather than harmful to certain viruses, and these therefore activate rather than inhibit autophagy-like processes (Figure 2). The small but diverse set of human tumor viruses contains examples for both autophagy-inhibitors and autophagy-inducers.

### 2.1. Viral Modulation of Autophagy-Mediated Immune Defense Mechanisms

It has been speculated that autophagy, an ancient mechanism that allows survival during nutrient deprivation, further developed during evolution to provide protection against additional forms of stress that multicellular organisms are exposed to, including infection by bacterial, protozoan and viral pathogens [121]. Research in recent years has provided conclusive evidence for autophagy playing a crucial role in host cell immunity [121]. Atg-proteins, however, may also function in immunity independently of their role in autophagy, as reported for ATG5 in macrophages infected with the protozoon *Toxoplasma gondii* [122]. 

In general, the regulation of autophagy (or of Atg-proteins) by immune signals is reciprocal and complex, and each can either induce or suppress the other [121]. Autophagy contributes to host defense in at least three ways (Figure 2). First, it targets pathogens for lysosomal degradation in a process that, more appropriately, is also referred to as xenophagy (eating of foreign matter) [123]. There is evidence that among the human tumor viruses, EBV is subject to xenophagy in epithelial cells [124]. 

Second, in the adaptive immune response, autophagy facilitates presentation of viral antigens on major histocompatibility complex (MHC) class II-molecules, which are usually loaded with antigen peptides derived from endocytosed pathogens [125,126,127]. Autophagy-mediated presentation on MHC class II has been originally described in lymphoblastoid cell lines (LCLs) for antigen peptides derived from the EBV nuclear antigen 1 (EBNA1) [125]. However, EBNA1-derived antigen processing via the autophagy-MHC class II-route appears to be epitope specific, and occurs only at a very low level. The latter may be due to the fact that EBNA1 localizes to the nucleus where it seems to be largely protected from autophagy [128]. Evidence for the *in vivo* importance of the autophagy-MHC class II pathway was provided by a study that demonstrated impaired MHC class II antigen-processing and ‑presentation in mice with dendritic cell specific knockout of the essential autophagy gene Atg5 [129]. A more recent study further suggests that viral antigens that are generated via an autophagy-like process can also be presented on MHC class I. However, this has only been reported during late stages of herpes simplex virus 1 (HSV-1) infection in macrophages [130].

**Figure 3:**
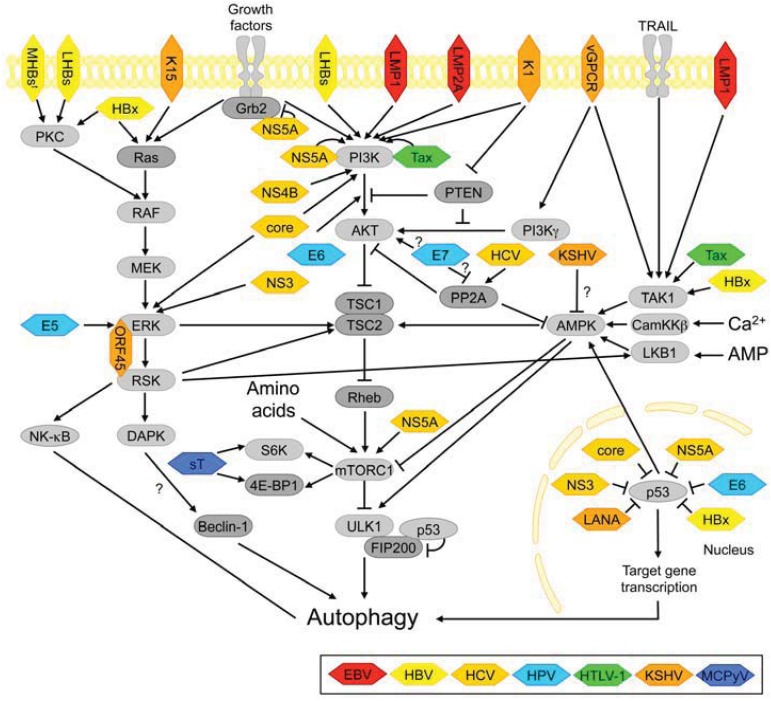
Autophagy regulation via nutrient- and growth factor sensitive signaling pathways and points of interference by viral proteins.

**Figure 4:**
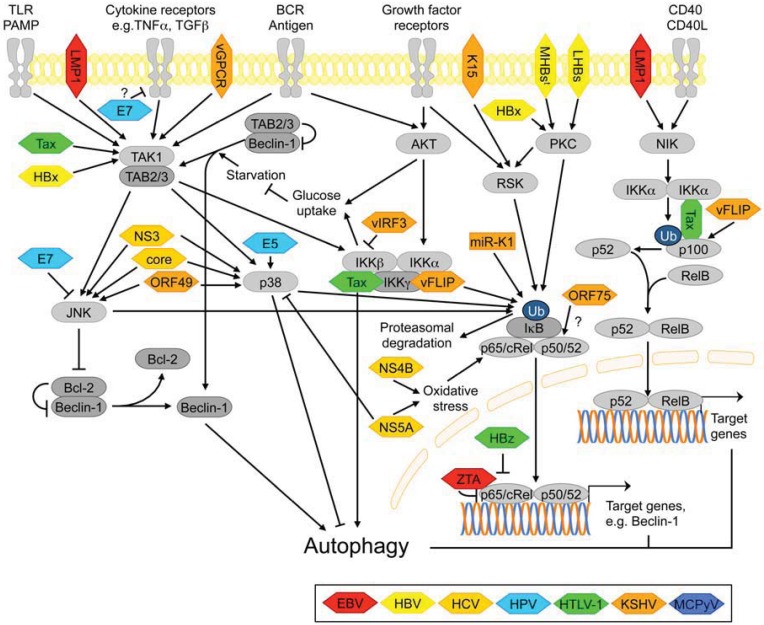
Autophagy regulation via the NF-κB pathway, and points of interference by viral proteins.

The eight established human tumor viruses interfere with a variety of host cell signaling pathways, including immune signaling pathways and pathways regulating cell growth and survival, often in response to nutrient availability (see Figures 3 and 4). See main text for details. Note: In this figure, and frequently in the literature, the term “autophagy” is used to generally denote biological processes that involve Atg-proteins, even if genuine autophagy (*i.e.*, degradation of intracellular macromolecules/structures) was not formally examined or if the late degradation steps in the pathway appeared to be blocked (for example in several studies on HBV and HCV modulation of autophagy, see main text for details). Light grey rounded rectangles-host cell kinases; dark grey rounded rectangles-other host cell proteins; colored hexagons-viruses/viral proteins.

Lastly, autophagy affects multiple innate immune signaling pathways. For example, autophagic vesicles in plasmacytoid dendritic cells can deliver cytosolic viral replication intermediates to acidified endosomes where they activate Toll-like receptor 7 (TLR7) and induce production of interferon α (IFNα) [131]. Conversely, TLR7 and various other TLRs, including TLRs 3 and 4 [132] as well as TLRs 1, 5 and 6 [133] have been reported to mediate autophagy induction in cultured macrophages when treated with the appropriate TLR-ligands. Some of the TLRs capable of signaling to the autophagy machinery also can be activated by some of the human tumor viruses including EBV, KSHV, HCV and HTLV-1 [134,135,136,137]. 

Another innate immunity signaling pathway triggered by viral infection comprises the double-stranded RNA sensing kinase PKR. PKR phosphorylates the α-subunit of the eukaryotic translation initiation factor 2 (eIF2α), which inhibits translation of host cell and viral mRNAs and stimulates autophagy [138]. The precise molecular events that lead to increased autophagy downstream of eIF2α are presently unknown [139]. PKR is clearly required for autophagy-induction in response to HSV-1 infection [140], yet, several other viruses also encode proteins that inhibit PKR signaling [141], including EBV BILF1 [12], KSHV viral interferon regulatory factors 2 and 3 (vIRF2/3) [98,99] and HCV non-structural protein 5A [52] (Figure 2). This supports the hypothesis that PKR plays an important role in virus-induced autophagy in general. 

While the studies cited above demonstrate a positive role for autophagy in host defense, two more recent reports provided evidence that HCV utilizes, rather than inhibits the autophagy machinery to quench an antiviral immune response [142,143]. In particular, HCV-infection and Atg-proteins were found to synergistically suppress expression of interferon β (IFNβ̣ or of IFN-inducible genes. These events are normally triggered upon exposure to HCV-derived pathogen associated molecular patterns [142], in cellular systems where HCV-infection increased autophagic markers [142,143] (Figure 2). It is unclear how inhibition of the IFN-regulated immune response by HCV-activated autophagy can be reconciled with the above mentioned predicted inhibition of PKR-induced autophagy by HCV NS5A [52], It is noted, that the literature on HCV and autophagy is particularly controversial in many respects. This may be due to the use of different cell lines/sublines and/or of different HCV-strains [144]. It is not clear, however, whether HCV genotypes indeed differ in their ability to induce autophagy [145] or not [55,146].

### 2.2. Virally Induced Autophagy and Replication

Some of the processes that are mediated by the autophagy machinery, such as antigen-processing or xenophagy, represent a threat to many pathogens, which, in turn, have adapted to this host response and in some cases, even found ways to take advantage of the Atg-proteins. A prominent example among the human tumor-associated viruses is provided by HCV. As discussed above, HCV may exploit the autophagy machinery to counteract an immune response [142,143]. Beyond that, multiple studies have reported that efficient HCV replication depends on Atg-proteins [55,142,146,147,148]. However, the various groups differ in their conclusions on the precise stage of infection during which Atg-proteins are required. Dreux *et al.* initially reported that replication efficiency in Huh7 cells was reduced when subgenomic HCV replicons were introduced after depletion of Atg-proteins [147]. Depletion of Atg-proteins in cells that already contained HCV subgenomic replicons, however, did not affect HCV RNA and protein levels. Thus, the authors concluded that the autophagy machinery is required for initiation of HCV replication but is dispensable once infection has been established [147]. A more recent study confirmed that Atg-proteins facilitate HCV replication upon initial infection [142], but there are conflicting reports on whether they are required for replication in infected cells [142,146,147,148]. Some studies imply that this is the case, since HCV viral particle release and survival of HCV-infected cells were decreased in the absence of Atg-proteins [142,143,145,148]. Clearly, additional studies are required to elucidate the basis for these discrepancies. 

How the autophagy machinery supports HCV replication is presently unclear. For several other positive-strand RNA-viruses, such as poliovirus, dengue virus and mouse hepatitis virus, which subvert the autophagy machinery to promote their life cycles, colocalization of viral proteins or RNA with markers of autophagy-related vesicles has been observed. This is consistent with the model that the membranes of these vesicles provide a scaffold for the viral replication complex [149,150,151,152,153]. However, in case of HCV, currently available data varies with regard to whether and in which stage of infection there is colocalization of viral replication factors with autophagosome-like vesicular membranes [55,147,148,154]. 

HBV replication also appears to depend on the autophagy machinery since replication was decreased when HBV-transfected Huh7.5-cells were treated with the autophagy inhibitor 3-methyladenine (3-MA) or when VPS34 or ATG7 were depleted [27]. Studies by the same group in HBV-transgenic mice with liver specific knockout of the critical autophagy-regulator Atg5 further suggest that the autophagy machinery is required for efficient HBV DNA replication *in vivo* [155]. In contrast, another group found that autophagy inhibition predominantly affected viral envelopment, rather than DNA replication [36]. Whether these different results are due to different sublines of Huh7 or different HBV strains used by the two groups, as suggested by Tian *et al.* remains to be determined.

Both HCC-associated viruses, HBV [27,36] and HCV [55,146], have been reported to induce autophagic markers in infected cells, however, without enhancing degradation of autophagy-substrates such as long lived proteins or p62. In case of HCV the initial reports cited above have been both substantiated and debated by more recent studies that provided evidence either for the incomplete autophagic response being due to impaired maturation of HCV-induced autophagosome-like vesicles [145] at late stages or for these vesicles actually being capable of mediating autophagic degradation of their cargo [142,156]. The study by Vescovo *et al.* suggests a partial explanation for this discrepancies: HCV-induced autophagy seems to predominantly target lipids, whereas turnover of organelles or proteins, which is commonly examined in autophagic flux assays [101] is largely unaffected by the virus.

In KSHV infection, viral particles are produced only in the lytic phase. Overexpression of the replication and transcription activator, RTA, the master regulator of the latent-to-lytic switch increases LC3 conversion and autophagic flux in 293T- and BJAB cells [87]. Conversely, treatment with the autophagy inhibitor 3-MA or Beclin-1 depletion inhibited expression of RTA-induced lytic genes, and diminished viral genome replication in a RTA-overexpressing PEL cell line. The mechanism(s) by which RTA increases autophagy and how autophagy subsequently facilitates lytic replication remain to be determined [87]. As discussed below, KSHV encodes two other proteins, vFLIP and vBcl-2 that inhibit autophagy. Since vFLIP is expressed during both the latent and lytic phases of the viral life cycle [157], one might speculate that vFLIP inhibition of autophagy contributes to maintenance of latency. VBcl-2 expression increases early in the lytic phase [158], and hence both vFLIP and vBcl-2 may serve to limit RTA-driven autophagy and lytic replication. In summary, KSHV may have developed multiple strategies to tightly control induction of lytic replication by modulating autophagy. 

## 3. Mechanisms of Viral Interference with Autophagy

As outlined above, modulation of autophagy apparently provides certain advantages to viral invaders. Yet, only very few viruses encode proteins that directly interact with components of the autophagy machinery (Figure 2). More commonly, viruses target autophagy-regulating upstream signaling pathways, including immune signaling pathways [141]. 

### 3.1. Viral Oncoproteins Directly Targeting the Autophagy Machinery

#### 3.1.1. Beclin-1—A Popular Target with Viral Proteins

Beclin-1 was discovered in a yeast 2-hybrid screen for interaction partners of the anti-apoptotic protein Bcl-2 [88]. Beclin-1 was the first mammalian Atg protein to be identified and the first Atg protein that was established as a tumor suppressor [118]. Binding of cellular Bcl-2 (cBcl-2) and of the viral Bcl-2 homologs encoded by KSHV (termed vBcl-2) [158,159] and murine γ-herpes virus 68, γHV68 (termed M11) [160] to Beclin-1 suppresses autophagy [89,161]. EBV also encodes two Bcl-2 homologs, BHRF1 [162] and BALF1 [163], but their potential interactions with Beclin-1 have not yet been examined. Upon starvation, cBcl-2 is phosphorylated by c-Jun N-terminal kinase 1 (JNK1), which causes disruption of the cBcl-2/Beclin-1 complex. In contrast, vBcl-2 lacks the relevant JNK phosphorylation sites [103] and, therefore, it constitutively associates with Beclin-1 to inhibit autophagy. It is interesting to note that several viral proteins including the EBV latent membrane protein 1 (LMP1) [13,14,15], KSHV ORF49 [85], HCV core and NS3, and HTLV-1 Tax [69] can activate the JNK signaling pathway, and this is predicted to activate Beclin-1 dependent autophagy. Conversely, expression of the HPV oncoprotein E7 can diminish JNK activation [67].

Another difference between cBcl-2 and vBcl-2 is that cBcl-2 disrupts the Beclin-1/UVRAG complex, whereas vBcl-2 does not, and instead forms a higher order Beclin-1/UVRAG/vBcl-2 complex [161]. These results suggest that cBcl-2 and vBcl-2 may inhibit Beclin-1 dependent autophagy by different mechanisms: cBcl-2 may function by preventing Beclin-1 from associating with VPS34, whereas the precise molecular events by which vBcl-2 inhibits autophagy remain to be determined. Regardless, however, the fact that Beclin-1 is an established tumor suppressor suggests that its inhibition by tumor virus proteins such as vBcl-2 importantly contributes to host cell transformation [141].

As indicated above, Beclin-1 is a common target among viruses that modulate autophagy. Other viral proteins that associate with Beclin-1 include the ICP34.5 neurovirulence protein of the α-herpesvirus HSV-1 [164], HIV-1 Nef [165] and the Influenza virus M2 protein [166]. While ICP34.5 association with Beclin-1 has been linked to inhibition of autophagy [167], it remains to be determined whether association of HIV-1 NEF or Influenza virus M2 with Beclin-1 inhibit autophagy.

#### 3.1.2. ATG3 Binding to FLIP-Proteins

In addition to inhibiting autophagy through formation of a vBcl2/Beclin-1 complex, KSHV also blocks this process via its FLICE-like inhibitor protein, vFLIP (encoded by ORF71/K13). vFLIP and its cellular counterpart cFLIP, inhibit death receptor-induced apoptosis [168,169] and also suppress starvation- or rapamycin induced formation of LC3-decorated vesicles and cell death associated with autophagy [90]. Of note, the anti-autophagic activity of vFLIP and cFLIP were independent of their anti-apoptotic activities, and both vFLIP and cFLIP were found to bind the autophagy-protein ATG3 competitively with LC3. For vFLIP, it was shown that the ability to interact with ATG3 was required for inhibition of cell death associated with autophagy [90].

#### 3.1.3. Interactions of Viral Proteins with Other Autophagy-Regulating Proteins

Biochemical evidence suggests interactions of various HBV- and HCV-proteins with components of the autophagy machinery. In particular, HBV X-protein (HBx) was shown to interact with VPS34 [27] and the small surface protein SHBs with LC3 [36]. In addition, HCV non-structural protein 3 (NS3) was found to associate and to colocalize with the immunity-associated GTPase family M protein (IRGM) [47], a known regulator of autophagy in response to bacterial infections [170]. NS4B coprecipitated with exogenous Rab5 and VPS34 [48] and NS5B was shown to coprecipitate with the ATG5-protein when overexpressed in yeast, and GFP-ATG5 and NS5B colocalized in Huh-7 cells [54]. Yet, several other groups working with various Huh-7 sub-lines infected with various HCV isolates observed little or no colocalization of various HCV-proteins such as core, NS3, NS4A/B and NS5A with autophagic marker proteins [55,145,146,148].

In addition to targeting autophagy regulators via protein-protein-interactions, HBV and HCV may also modulate autophagy through transcriptional activation of Beclin-1 expression. While in case of HBV, reporter assays suggested that HBx may transactivate the Beclin-1 promoter [26], mechanistic details of how HCV increased Beclin-1 expression remain to be determined [55]. 

### 3.2. Autophagy-Regulating Signaling Pathways Targeted by Viral Oncoproteins

Autophagy is activated above basal levels in response to diverse environmental and physiological stressors such as nutrient- or growth factor deprivation, hypoxia, ER- and redox stress, organelle damage or pathogen infection [121]. This implies that autophagy is tightly connected to cellular metabolism and to diverse stress-sensitive signaling pathways. Yet, the precise molecular links between these pathways and the autophagy machinery have not yet been fully elucidated. In addition, virtually all the major cellular stress-sensing signaling pathways have been implicated in human cancer, and most of these pathways are also modulated by tumor viruses. In this section, we describe how viruses disturb some of the central stress-sensing host cell signaling pathways and discuss potential effects on autophagy.

#### 3.2.1. PI3K-AKT and mTORC1 Signaling

The PI3K-AKT signaling pathway is activated downstream of growth factor receptors such as insulin-receptor and epidermal growth factor receptor (EGFR) and regulates several key aspects of cell physiology including cell cycle, metabolism and apoptosis (Figure 3). Activation of PI3K-AKT signaling promotes cell growth, proliferation and survival [171]. A central mediator of this pathway with regard to cell growth is the (m)TOR-complex 1 (mTORC1), a multiprotein complex comprising the Ser/Thr kinase mammalian target of rapamycin (mTOR) and regulatory proteins Raptor, GβL, PRAS40 and Deptor (reviewed in [104]). ERK signaling, decreased AMPK signaling and availability of amino acids also activate mTORC1 (see below). mTORC1 supports cell growth by activating mRNA translation and ribosome biogenesis and by inhibiting autophagy [104]. In fact, inhibition of mTORC1 and its counterparts in other organisms is sufficient to activate autophagy even in the presence of nutrients [172]. Although TOR has long been known to be a key suppressor of autophagy [172], the mechanisms by which it regulates this process in mammalian cells have much more recently been delineated and the autophagy-initiating kinase ULK1 was identified as an mTORC1-substrate (for review, see [116] (Figure 3). While mTOR is rarely mutated in human cancers [173], alterations in PI3K-AKT signaling are among the most frequent alterations observed in a wide variety of tumors [174]. Such cancers are expected to have dysregulated mTORC1 signaling and dysregulated autophagy. Autophagy dysregulation by this pathway may be common in virus-associated malignancies, too. Even though the mTOR kinase itself does not seem to be a direct target of any viral protein, all the known human tumor viruses appear to interfere with PI3K-AKT-mTOR signaling, most likely to exploit the growth- and survival promoting function of these pathways. Tumors with hyperactive mTORC1 may be sensitive to treatment with mTOR(C1)-inhibitors such as rapamycin, and indeed, such drugs have been suggested for treating infections and/or tumors caused by EBV [175], KSHV [176,177], HBV [178], HCV [179], HPV [180] and HTLV-1 [181,182]. 

EBV LMP1 mimics a ligand-independent, constitutively active CD40 receptor [183] and is sufficient for transformation of rodent cells and primary B lymphocytes *in vitro* [184,185] and *in vivo* [186]. LMP1 was shown to activate the PI3K-mTOR pathway in B cell lines [21] and LMP1-expression was positively correlated with phosphorylation of mTOR and its substrates ribosomal protein S6 kinase (S6K) and eukaryotic translation initiation factor 4E-binding protein (4E-BP1) in nasopharyngeal carcinoma patient samples [20]. The second EBV encoded transmembrane protein, LMP2 [187], or more precisely, the LMP2A splice variant, is dispensable for B cell immortalization *in vitro* [188], but it appears to enhance LMP1’s ability to promote carcinogenesis in a transgenic mouse model [189]. LMP2A was shown to activate PI3K-AKT- and mTOR signaling in several cell lines, including a nasopharyngeal carcinoma line [22,23,24], and *in vivo*, in B-cells of LMP2 transgenic mice [190]. The LMP2B splice variant lacks an N-terminal cytoplasmic domain and hence important signaling functions seen for LMP2A [191,192,193]. 

Multiple KSHV-proteins have been implicated in activation of PI3K-, AKT- and/or mTOR signaling. In particular, the viral G-protein coupled receptor homolog (vGPCR), a lytic gene, signals to mTORC1 via PI3Kγ [97], a PI3K isoform that is mainly expressed in white blood and endothelial cells and that is uniquely activated by GPCRs [97,194]. vGPCR signaling to mTORC1 drives endothelial cell proliferation [195] and mTORC1 signaling may in turn promote expression of the latent-to-lytic switch master regulator RTA [176]. The latter results may require further confirmation since the study is limited to pharmacologic inhibition of mTORC1 with rapamycin in one particular PEL cell line where rapamycin did not induce growth arrest. Moreover, the finding that mTORC1 is a positive regulator of RTA-driven lytic activation cannot be easily reconciled with another report discussed above, that provided evidence for autophagy promoting KSHV lytic replication, since autophagy is inhibited by mTORC1 [87]. Thus, additional studies are required to elucidate the regulatory connections between mTORC1, RTA and autophagy. In addition, KSHV activates PI3K-, AKT- and mTOR signaling in both B lymphocytes [78] and endothelial cells [79,80] via its K1 protein, a constitutively active B-cell receptor homolog which is predominantly expressed during lytic replication [196,197,198]. 

This figure gives a simplified overview of the cell’s nutrient and growth-factor regulated cellular signaling pathways: (1) the PI3K pathway and its important signaling mediators AKT, the TSC1/2 GTPase activating complex for the GTPase Rheb, which activates mTORC1, and mTORC1 (note: individual complex proteins have been omitted for clarity); (2) the RAF-MEK-ERK MAPK-module and (3) the AMPK pathway. Interactions of these pathways with the p53 tumor suppressor, which is commonly inactivated by viral oncoproteins are also shown. These pathways are directly connected to the autophagy machinery via the ULK1-kinase complex and Beclin-1. See main text for additional details. See Figure 4 for AKT- and TAK1 regulation of NF-κB signaling, regulation of Beclin-1 by JNK signaling and viral modulators of the JNK pathway, as well as additional upstream regulators, downstream mediators and viral modulators of TAK1 signaling. Mechanistic details on how viral proteins interfere with nutrient- and growth factor-regulated signaling pathways are discussed in the text, except for the following: HBV LHBs and MHBs^t^ signal to RAF via protein kinase C (PKC) [31]. HBx can also activate PKC [199]. HCV NS5A does not directly target mTOR but its regulator FKBP38 [200] and may inhibit ERK signaling by interacting with GRB2 [50,51]. Question marks indicate that it is currently unclear whether DAPK-phosphorylation of Beclin-1 occurs physiologically, which HCV-protein is involved in inhibition of PP2A, which KSHV protein is involved in inhibition of AMPK, and whether HPV E7 activates AKT via an retinoblastoma protein dependent or -independent mechanism involving PP2A [62,63]. Light grey rounded rectangles-host cell kinases; light grey ovals-host cell transcription factors; dark grey rounded rectangles-other host cell proteins; colored hexagons-viral proteins; : activation; to ⊥: inhibition. -: no general consensus in the literature on activation/inhibition, outcome may be cell type specific.

Modulation of PI3K-AKT-mTOR signaling may also play an important role in HBV-associated liver carcinogenesis. Studies in cultured cells and in clinical samples collectively suggest a model whereby PI3K-AKT-mTOR signaling is initially activated by the HBV large surface protein LHBs, whereas at later stages, PI3K-AKT-mTOR signaling suppresses LHBs-expression and HBV-replication [32,33,34,201].

The situation is similar in HCV infection, in that mTOR signaling suppresses viral replication, even though the virus can increase mTOR activity [202,203,204]. The NS5A protein may play an important role in this, since it has been reported to activate mTORC1 signaling via both PI3K-AKT-dependent [49,50,205] and independent mechanisms [200]. On the other hand, NS5A was also found to be phosphorylated in a rapamycin-sensitive manner when ectopically expressed in COS-1 cells, suggesting that it functions downstream of mTORC1 [206]. Other HCV proteins activating AKT signaling include NS4B and core [46]. It is interesting to note that the group that reported that NS4B is sufficient to induce autophagic markers did not observe alterations in mTORC1-activity by NS4B [48]. 

In cells expressing the HPV oncoprotein E6, mTORC1-activity is sustained even under conditions of growth factor deprivation [58,59]. Although controversial reports exist in the literature regarding the underlying mechanism [59,207], studies conducted in a physiologically relevant cellular system, human foreskin keratinocytes (HFKs), suggest that this is due to E6 sustaining the activity of AKT, which stimulates mTORC1 [59]. Whether this restrains autophagy-induction under these conditions remains to be investigated. The second HPV oncoprotein, E7 also has been reported to activate AKT signaling in several cell types including HFKs [62,63]. Although this is predicted to inhibit autophagy, E7 was found to induce an autophagy-like process in normal human keratinocytes even when the cells were grown in normal medium [68]. Additional studies are required to define the mechanism by which HPV E7 expression activates the autophagy machinery, although it has been speculated that it may arise as a consequence of metabolic stress [208], potentially due to induction of the Warburg effect by the HPV E7 protein [209] and the concomitant decrease in ATP production. Moreover, it will be interesting to investigate if autophagy is deregulated in cervical cancer cells, which co-express the E6 and E7 oncoproteins that may have opposing effects on autophagy-like processes. 

Similar to HPV E6, the HTLV-1 oncoprotein Tax allows for sustained AKT phosphorylation under low serum conditions, and this may be due to Tax interacting with the regulatory p85 subunit of PI3K [70]. Another study showed that Tax-conferred growth factor independence through mTORC1 [71].

Currently, there are no reports in the literature that specifically address interference with autophagy by the most recently discovered tumor virus, MCPyV. Yet, the MCPyV small T-antigen, which is regularly expressed in MCPyV-positive tumors [75] was shown to increase phosphorylation of the two mTORC1 effectors and substrates, 4E-BP1 and S6K, potentially by inhibiting their dephosphorylation [75]. It is interesting to note that in another recent study, activating mutations in PI3K were detected almost exclusively in MCPyV negative Merkel cell carcinoma specimens [210]. This underscores the importance of PI3K signaling and its downstream targets such as mTORC1 for MCC development and supports the model that MCPyV has evolved strategies to activate this critical pathway.

#### 3.2.2. ERK Signaling

The ERK pathway represents the prototypical example of a mitogen-activated protein kinase (MAPK) cascade where ERK (a MAPK) is activated by MEK, a MAPK kinase (MAPKK), which in turn is activated by RAF, a MAPKK kinase (MAPKKK). RAF is typically activated by the small GTPase Ras downstream of receptor tyrosine protein kinases [211] (Figure 3). Many effects of ERK signaling are cell type and context dependent [212], and this may include its role in autophagy regulation. ERK signaling contributes to mTORC1 activation, which suppresses autophagy [104]. In addition, at least in certain cell types, the ERK-substrate 90 kD ribosomal protein S6 kinase (RSK) can inhibit death-associated protein kinase (DAPK), a potential positive regulator of Beclin-1 dependent autophagy [213,214], and both ERK and RSK can inhibit the Ser/Thr-kinase LKB1 [215] and thus interfere with activation of the pro-autophagic kinase AMPK (see below) [216]. Finally, ERK may modulate autophagy via RSK-dependent activation of NF-κB signaling [217], which is discussed in more detail below.

Tumor viruses that interfere with ERK signaling include KSHV, HBV, HCV and HPV. KSHV modulation of ERK signaling has been studied in the context of lytic gene regulation. KSHV ORF45, an immediate early gene expressed during primary infection as well as reactivation [218] was shown to promote lytic gene expression by recruiting ERK and its substrate RSK into a common complex and by sustaining the enzymatic activity of both kinases [219]. In addition, several splice variants of the transmembrane protein K15 were also reported to activate the ERK pathway [81]. HBV activates the Ras-RAF-MEK-ERK pathway via HBx and the large and middle surface antigens [30,31,32,51,61,220,221,222] and HPV via its E6 and E7 oncoproteins [61]. For HCV, evidence has been provided that core and NS3 activate ERK signaling [37,38] while NS5A may suppress this pathway [50,51]. For any of the viruses discussed here, KSHV, HBV, HCV, and HPV, it remains to be examined whether their interference with ERK signaling affects autophagy. 

#### 3.2.3. AMPK-Signaling

The AMP-activated protein kinase, AMPK, is a major cellular energy sensor and may positively regulate autophagy by inhibiting mTORC1 (for reviews, see [104,216]). However, several studies demonstrated that AMPK can also directly target the autophagy machinery. AMPK associates with and phosphorylates the autophagy-initiating kinase ULK1 [223,224,225,226] (Figure 3). AMPK-dependent phosphorylation of ULK1 may be important for autophagosome biogenesis since it is required for correct intracellular localization of mAtg9, a putative membrane carrier protein [227,228]. AMPK activity is modulated by the cellular ATP/AMP ratio and requires phosphorylation by an upstream kinase, such as LKB1, CamKKβ or potentially TAK1, which also plays an important role in NF-κB activation [229]. All three of these kinases have been reported to mediate autophagy via AMPK, at least in specific cell lines and contexts [230,231,232]. Moreover, AMPK-dependent autophagy induction seems to require p53, which, as discussed below, is commonly inactivated by tumor viruses [233]. 

Reports explicitly connecting AMPK or one of its upstream kinases to human tumor viruses and autophagy are scarce in the literature. Expression of simian vacuolating virus 40 (SV40) small T antigen has been demonstrated to inhibit cell death in glucose deprived cultured cells by activating AMPK and increasing autophagy [234]. However, whether small T antigens of other polyomaviruses, such as that of the carcinogenic MCPyV can also activate AMPK remains to be determined. A recent study detected no evidence for AMPK activation in EBV-positive B cells that underwent autophagy in response to NF-κB inhibition [16], yet it cannot be ruled out that AMPK may still be important in this setting, since even basal activity of AMPK is sufficient for induction of autophagy [235,236]. 

However, beyond the specific context of autophagy, there is increasing evidence for crosstalk between AMPK signaling and viral infection, including infection by tumor viruses [237]. In particular, AMPK-inhibition was observed in KSHV-infected endothelial cells [79]. Moreover, in Huh-7 cells, HCV infection or the presence of a subgenomic replicon decreased AMPK-activity [238]. Conversely, pharmacologic AMPK activation blocked viral replication [238]. Since AMPK is an inhibitor of lipid biosynthesis, AMPK activation may block formation of a membranous web that is critically important for HCV replication [216,239,240]. However, since HCV induces, rather than inhibits, formation of autophagic vesicles [48,55,142,146,147,156], it remains to be determined whether and under which conditions HCV-modulation of AMPK affects autophagy in infected cells.

Given that AMPK- and AKT-mTOR signaling have opposing effects on cell physiology, some findings on the biochemical and biological activities of these pathways in the context of HCV-infection cannot be easily reconciled. AMPK activation, for instance, which decreases HCV-replication, should result in mTOR-inhibition. Yet, as was pointed out above, mTOR is also a negative regulator of HCV-replication, and its inhibition should favor HCV replication. This suggests that AMPK and mTOR inhibit HCV-replication through different molecular mechanisms and that maximum replication should occur when both AMPK- and mTOR signaling are suppressed. Interestingly, a study that investigated biopsies of chronically hepatitis C infected liver tissues showed increased expression of protein phosphatase 2A (PP2A), which negatively regulates both the AMPK- and the AKT-mTOR pathway in cultured cells expressing HCV proteins [241]. Clearly, additional studies are required to further characterize the profound HCV-induced changes in the host cell signaling pathways regulating cell growth, metabolism and autophagy that are suggested by the currently available data. 

Several viral oncoproteins such as EBV LMP1 [15], KSHV vGPCR [242], HBx [243] and HTLV-1 Tax [69] were shown to signal through the potential AMPK-activating kinase TAK1. TAK1-AMPK connections in the context of these viral proteins, however, have not been examined. 

#### 3.2.4. Other Kinases Involved in Starvation-Induced Autophagy

Kinases other than AMPK and mTOR have also been implicated in starvation-induced autophagy. Positive regulators include the IKK kinase complex (discussed in greater detail below), which was found to be important for efficient upregulation of autophagy in response to starvation [244] and JNK1 (see above), which upon starvation releases Beclin-1 from an inhibitory complex with Bcl-2 [103]. The p38α kinase is a negative regulator of both basal and starvation induced autophagy and modulates trafficking of the putative membrane carrier protein mAtg9 [102]. Viral oncoproteins upregulating p38 signaling include EBV LMP1 [17,21], KSHV vGPCR [86], K15 [81] and ORF49 [85], the HBV protein HBx [29], HCV core and NS3 [37,38], the HPV E5 protein [56] and HTLV-1 Tax [69]. Conversely, HCV NS5A inhibited p38-activity [53]. JNK and p38 may also modulate autophagy through activating NF-κB (see below). Lastly there is evidence from studies in yeast which require further confirmation in mammalian cells that PKR, a kinase targeted by many viruses because of its function in antiviral immune signaling (see above) may also play a role in starvation-induced autophagy [140]. 

#### 3.2.5. The p53 Tumor Suppressor

The p53 tumor suppressor also has an important role in controlling metabolic stress [245]. In response to various autophagy-inducing stimuli, including nutrient-deprivation and mTOR-inhibition, nuclear p53 induces transcription of genes that positively regulate autophagy such as the lysosomal protein Damage-Regulated Autophagy Modulator (DRAM) [246] and Sestrin 2 [247,248]. Moreover, p53 transcriptionally activates negative regulators of the PI3K-AKT-mTOR signaling pathway such as AMPKβ, TSC2, PTEN and IGF-BP3, at least in certain cell types and in response to particular stressors [249]. A notable exception to the general trend that p53-target genes promote autophagy is the TP53-Induced Glycolysis and Apoptosis Regulator (TIGAR), a fructose-2,6-bisphosphatase [250] that inhibits autophagy by decreasing ROS-levels under conditions of nutrient starvation or metabolic stress [251]. In contrast to nuclear p53, cytoplasmic p53 mutants inhibit autophagy by interacting with FIP200, a component of the autophagy-initiating ULK1-kinase complex [252,253]. 

Various viral oncoproteins have been reported to interfere with p53 function but whether this contributes to autophagy modulation by any of the human tumor viruses has not been examined yet. The most prominent examples include SV40 large T antigen, Adenovirus E1b and HPV E6, which all form complexes with p53 and functionally inactivate it (reviewed in [254]). In particular, HPV E6 recruits the host cell encoded E3 ubiquitin ligase UBE3A (E6AP) to target p53 for ubiquitination and proteasomal degradation [60,255,256]. The immediate benefit of E6-mediated p53-inactivation for the virus presumably consists in abrogation of the p53-dependent apoptotic response which otherwise would be triggered to counteract excessive cell proliferation driven by E7 [5]. In addition, KSHV latency associated nuclear antigen (LANA) has been reported to interact with p53 and inhibit its transcriptional activity [82]. The HBx protein was reported to interact with p53 in HBV-positive HCC tissue lysates [28], and there have also been reports for interactions of the HCV proteins core [42,43,45], NS3 [41] and NS5A with p53 [44]. HTLV-1 Tax also inhibits p53 transcriptional activity [73,257,258,259]. Even though p53 was originally discovered through its interaction with SV40 large T antigen [257] there is no evidence that MCPyV large T antigen associates with p53.

#### 3.2.6. NF-κB Signaling

Nuclear factor κB transcription factors are executing the biological activities of multiple stress-sensitive signaling pathways, including immune signaling pathways (Figure 4). Not surprisingly, they regulate expression of a broad variety of genes related to immunity, cell proliferation, differentiation and survival [139,229]. An overview of the NF-κB signaling network as it pertains to autophagy is given in Figure 4.

There is complex and reciprocal regulation of NF-κB signaling and autophagy and both activation and suppression of one by the other have been observed. Many of the reported effects, however, may be specific to the cell type and/or the autophagy-modulating treatment applied. For example, in HTLV-1 transformed cells and ATL cell lines, autophagy induced by Geldanamycin treatment inhibited NF-κB signaling by selectively degrading IKK and NIK [260,261]. On the other hand, in mouse embryonic fibroblasts (MEFs), Atg-proteins ATG5, ATG7, Beclin-1 and VPS34 were required for activation of NF-κB by tumor necrosis factor-α (TNFα) [262]. Conversely, the NF-κB family member p65 positively regulated basal autophagy by transactivating the Beclin-1 promoter in T-cells [263]. However, NF-κB suppressed TNFα-, but not starvation-induced autophagy in Ewing sarcoma‑cells [264]. Finally, a recent study suggested that NF-κB signaling can be activated in parallel with autophagy by pro-autophagic stimuli via a mechanism that involves the TAK1-binding proteins 2 and 3 (TAB2/3) switching from binding and inhibiting Beclin-1 under basal conditions to associating and activating TAK1 [265]. Moreover, the NF-κB-regulating kinase IKKβ can promote autophagy independently of the transcription factor NF-κB. AMPK and JNK appeared to be important downstream mediators of IKK in this context [244]. Conversely, in B cell lymphomas, IKK inhibited autophagy as a consequence of promoting glucose uptake via parallel activation of NF-κB and AKT [16]. 

This figure gives a simplified overview of the cellular signaling pathways that regulate NF-κB transcription factors. These transcription factors function as homo- or heterodimers formed by the five proteins of the NF-κB/Rel-family: NF-κB1/p50, which is generated by proteolytic cleavage of its precursor p105 (omitted for clarity), NF-κB2/p52 generated by cleavage of the precursor p100, RelA/p65, RelB and c-Rel. The canonical NF-κB pathway is triggered by B-cell-, T-cell, cytokine-, growth factor- or Toll-like receptor signaling, which lead to activation of the inhibitor of κB kinase (IKK) complex in the cytoplasm. This complex consists of the catalytic subunits IKKα and IKKβ and the negative regulatory subunit IKKγ/NEMO (NF-κB essential modulator). IKK activation often also requires the transforming growth factor β-activated protein kinase 1 (TAK1). IKK complexes phosphorylate an inhibitor of κB (IκB) protein, which binds NF-κB dimers and retains them in the cytoplasm. Phosphorylation by IKKβ induces ubiquitination and proteasomal degradation of IκBs and releases NF-κB dimers, which subsequently undergo additional phosphorylation and acetylation events (omitted for clarity) and translocate to the nucleus to activate transcription of their target genes. Non-canonical NF-κB activation involves NF-κB-inducible kinase (NIK) mediated activation of IKKα homodimers, which then phosphorylate the NF-κB2/p100 precursor. This allows proteolytic processing of p100 to p52 which associates with RelB to form a functional transcription factor complex that enters the nucleus [229]. See main text for additional details and Figure 3 for additional autophagy-regulating pathways downstream of AKT and TAK1 and for upstream regulation of RSK. Specific tumor virus derived surface antigens/pathogen associate molecular patterns (PAMPs) activating the immune receptors shown in this figure have been omitted for clarity. Mechanistic details on how viral proteins interfere with NF-κB signaling have been included and are discussed in the text, except for the following: HTLV-1 Tax and KSHV vFLIP interact with IKKγ [266,267,268,269,270] and stimulate p100 expression and processing independently of NIK, whereas Tax may also directly interact with IKKα and p100 [271,272,273]. Question marks indicate that it is not clear at the moment where HPV E7 interferes with TNFα- and TGFβ-receptor signaling, and by which mechanism KSHV ORF75 activates NF-κB-dependent transcription. Light grey rounded rectangles-host cell kinases; light grey ovals-host cell transcription factors; dark grey rounded rectangles-other host cell proteins; colored hexagons-viral proteins; colored box-viral microRNA; : activation; to ⊥: inhibition. -: no general consensus in the literature on activation/inhibition; outcome may be cell type specific.

The NF-κB pathway is a common target of several of the human tumor viruses. NF-κB signaling plays a central role throughout the entire life cycle of the γ-herpesviruses EBV and KSHV and is constitutively activated during latency [274]. This is largely due to the action of LMP1 and vFLIP, in EBV- and KSHV infected cells, respectively [92,274,275]. LMP1 activation of NF-κB signaling was shown to suppress autophagy in B cells, a natural host of EBV [16], while vFLIP was shown to inhibit autophagy by targeting ATG3 [90] (see above). So far, no studies have implicated activation of NF-κB signaling in autophagy regulation by vFLIP. EBV and KSHV each express additional regulators of NF-κB signaling [274]. The EBV immediate early protein ZTA, which is involved in the latent-to-lytic switch, inhibits the transcriptional activity of p65 [25], while KSHV microRNA miR-K1 and the vGPCR enhance NF-κB activity [91,93,94,95]. In addition, ORF75 and K15 may activate NF-κB signaling and vIRF3 acts as a suppressor [93,100]. The effect of KSHV K1 on NF-κB activity is controversial in the literature [93,100]

There is evidence that HBV HBx and a truncated form of the middle surface antigen (MHBs^t^) can activate NF-κB-dependent transcription, and this may contribute to enhanced tumor burden in MHBs^t^ transgenic mice [31,35]. In the context of HCV infection, NF-κB, along with p38, JNK and ERK1/2 is activated as a consequence of ROS production in tissue culture cells [276]. This causes increased production of TGFβ1 [276], a cytokine that promotes autophagy in HCC cell lines [277]. Evidence has been provided for NS4B [40] and NS5A [39], respectively, being important for the induction of oxidative stress by HCV. Additional HCV proteins shown to activate NF-κB, potentially via the JNK- and p38 pathways include core and NS3 [37]. There is no evidence that HBV and HCV modulate autophagy through these pathways.

HPV positive cervical cancer cells are resistant to cytostatic effects of TNFα [64] and TGFβ [65], which both act via NF-κB and can activate autophagy. The E7 oncoprotein was shown to mediate the resistance to TNFα and TGFβ-induced growth inhibition [64,65], and to suppress TNFα-induced NF-κB-activation when expressed in cervical epithelial cells [66]. Whether these effects of E7 are associated with altered host cell autophagy has not been determined. 

In HTLV-1 infected cells, NF-κB is constitutively activated, predominantly via the non-canonical pathway. Even though the Tax oncoprotein upregulates both canonical and non-canonical NF-κB signaling [74,76], the HTLV-1 encoded basic leucine zipper transcription factor HBZ selectively interferes with the canonical pathway [77]. As described in a previous paragraph, autophagy was found to inhibit NF-κB signaling when HTLV-1 transformed cells and ATL-cell lines were treated with Geldanamycin, and this was suggested as a potential therapeutic strategy for ATL [278].

#### 3.2.7. Signaling Pathways Activated by ER Stress

**Figure 5:**
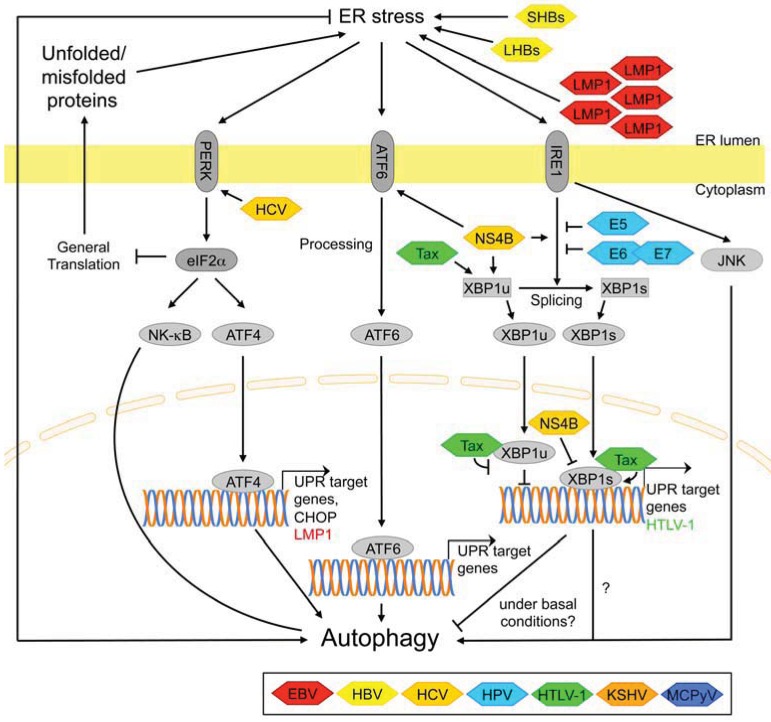
Autophagy regulation by ER stress/UPR signaling pathways and points of interference by viral proteins.

The endoplasmic reticulum (ER) is the major cellular site for folding and maturation of secreted and transmembrane proteins. ER stress ensues when the number of unfolded and/or misfolded proteins that enter the ER exceeds the capacity of the ER chaperone machinery and triggers a regulatory response termed the unfolded protein response (UPR) that adjusts the ER work load to its folding/refolding capacity [279]. Moreover, the UPR also activates autophagy [139]. The signaling pathways that activate the UPR have been reviewed in detail elsewhere [279,280] and are outlined in Figure 5.

Ectopic expression of the EBV oncoprotein LMP1 causes ER stress and activates all three arms of the UPR [19]. Conversely, PERK-dependent phosphorylation of elF2α and activation of ATF4 increased LMP1 expression [19]. It is interesting to note that LMP1, when overexpressed at low levels, activates NF-κB signaling rather than the UPR [19,281]. Importantly, however, vast variations in LMP1 levels spanning up to two orders of magnitude have been observed in EBV-infected clonal lymphoblast populations [282]. Moreover, induction of NF-κB- and of PERK-elF2α signaling by LMP1 are independent and require different domains of LMP1 [19,282]. Interestingly, LMP1 also induces autophagy in a dose-dependent manner via the same domains that are also involved in UPR-activation, and autophagy mediates LMP1 degradation [18]. Thus, it is tempting to speculate that in fact ER stress caused by high levels of LMP1 is the trigger for autophagy upregulation [283]. Therefore, this pro-autophagic function of LMP1 when expressed at high levels is not inconsistent with a more recent study that reported suppression of autophagy by LMP1 signaling through IKKβ/NF-κB due to increased capacity for glucose uptake [16]. Whether the second oncogenic γ-herpesvirus, KSHV, also modulates ER stress and the UPR is not known at present.

Ectopic expression of the HBV small surface protein SHBs was reported to be sufficient for the induction of autophagic markers, and the underlying mechanism involved upregulation of all three arms of the UPR [36]. In addition, mutant versions of the large surface protein LHBs were reported to induce ER stress, however, a potential effect on autophagy was not explicitly examined [33]. 

The unfolded protein response (UPR) is triggered by endoplasmic reticulum (ER) stress and comprises three branches, each of which involves an ER transmembrane protein that senses the protein folding status in the ER and transduces this information to the cytosol. In the first branch, upon ER stress, the bifunctional inositol requiring enzyme 1 (IRE1) oligomerizes and autophosphorylates and thereby activates its endoribonuclease activity. This allows for splicing of an mRNA that encodes the transcription factor X-box binding protein 1 (XBP1). The product of the spliced XBP1-mRNA (XBP1s), in turn, upregulates expression of UPR target genes, including chaperones and ER-associated degradation (ERAD) proteins. The product of the unspliced XBP1-mRNA (XBP1u) acts as a transcriptional repressor. In the second branch, ER stress triggers processing of activating transcription factor 6 (ATF6), which spans the ER membrane as an inactive precursor. Subsequently, the cytosolic ATF6 fragment enters the nucleus and activates expression of UPR target genes. In the third branch, ER stress activates PKR-like eIF2α kinase (PERK), which phosphorylates eIF2α and shuts down mRNA translation to reduce the number of proteins entering the ER. In parallel, PERK selectively promotes translation of certain stress-associated factors such as the ATF4 transcription factor. IRE1-, ATF6- and PERK signaling are activated independently but mutually reinforce each other, resulting in partially overlapping sets of UPR target genes. These UPR target genes include components of the autophagy machinery, and activation of autophagy allows degradation of ER domains that contain misfolded and aggregated proteins [284,285]. Note that eIF2α phosphorylation at the PERK-target site, S51, is also required for autophagy in response to other stressors, including viral infection, when eIF2α Ser51 is phosphorylated by PKR [140]. The molecular events that cause induction of autophagy downstream of eIF2α phosphorylation are incompletely understood, with the exception of the above mentioned transcriptional effects on autophagy genes [139]. Additional mechanisms of autophagy induction by ER stress may include IRE1 signaling to JNK [286], a known kinase of Beclin-1, and PERK-mediated NF-κB activation [287,288]. See Figure 4 for additional host cell signaling pathways and viral proteins modulating NF-κB and JNK signaling, and events downstream of JNK-activation that contribute to autophagy-modulation. Mechanistic details on how viral proteins interfere with UPR signaling are discussed in the text. The question marks indicate that the finding of XBP1 increasing autophagy under basal conditions so far is limited to neurons [289]. This implies that XBP1 suppresses autophagy and gives rise to the hypothesis that the XBP1 branch of the UPR functions to prevent excessive autophagy upon ER stress. Additional studies are required to test this possibility [289,290] and to clarify whether and under which conditions XBP1 activates or inhibits autophagy. Light grey rounded rectangles-host cell kinases; light grey ovals-host cell transcription factors; light grey boxes-host cell mRNAs; dark grey rounded rectangles-other host cell proteins; colored hexagons-viral proteins; : activation; to ⊥: inhibition. -: no general consensus in the literature on activation/inhibition; outcome may be cell type specific.

Multiple *in vitro* [48,142,146,291,292] and *in vivo* [293,294] studies collectively suggest that HCV-infection activates upstream regulators in all three branches of the UPR, but this does not lead to the induction of UPR-responsive genes [291,292,293,294]. Mechanistically, at least in the XBP1-branch of the UPR, the NS4B protein may be responsible for increasing XBP1-mRNA levels and splicing, but also for suppressing the transcriptional activator function of spliced XBP1 [291,292]. However, two studies provide evidence that the activators of the three UPR signaling pathways, PERK, ATF6 and IRE1, as well as a PERK-target gene, the transcription factor C/EBP-homologous protein (CHOP), play an important role upstream of the HCV-induced autophagy-like process in promoting viral replication [142,146] and in suppressing the antiviral immune response [142]. Thus, additional studies are required to elucidate how HCV perturbs UPR signaling and autophagy in infected cells.

Little is known regarding modulation of ER-stress pathways by high-risk HPV proteins. There is evidence that in human keratinocytes, E5, and also E6/E7 suppress activation of the ER stress factor XBP1 under basal conditions, *i.e.*, in the absence of ER- or other forms of stress [57]. Of note, XBP1 appears to suppress autophagy under these conditions, at least in neurons, giving rise to the hypothesis that suppression of XBP1 signaling by HPV-proteins increases basal autophagy [289,290]. Although basal autophagy levels may indeed be elevated in HPV positive cells [68], additional studies are required to clarify the relationship between HPV proteins, ER stress signaling pathways and autophagy.

Conversely, there is evidence that HTLV-1 via its Tax-protein upregulates XBP1 transcription (but not splicing) in unstressed cells, and *vice versa*. The mechanism may involve physical interaction between Tax and the protein products of both the unspliced and the spliced XBP1-mRNAs [72]. While Tax expression increased UPR signaling under basal conditions, ER stress did not increase HTLV1-expression despite increasing XBP1-transcription and splicing. It remains to be determined whether the Tax-XBP1-interaction affects basal- or stress-induced autophagy in HTLV1-infected cells. 

## 4. Concluding Remarks

By mediating turnover and recycling of macromolecular and supramolecular intracellular structures, autophagy plays a central role in maintaining cellular homeostasis under physiologic conditions as well as under conditions of stress. Accordingly, this process is regulated by virtually all cellular signaling pathways that sense cell-intrinsic or environmental perturbations. These networks include immune signaling pathways and indeed, autophagy is emerging as an important defense mechanism against pathogens, including viruses. A small subset of viruses is known to cause or at least contribute to a variety of human cancers and a large number of studies support the notion that infection with these viruses disturbs signaling networks far beyond pathways primarily associated with immunity. Indeed, signaling pathways that primarily control cell growth, proliferation and survival, *i.e.*, processes that are generally deregulated in cancer cells, are affected as well. These pathways are also connected to regulation of autophagy. With exception of the most recently discovered tumor virus, MCPyV, there is evidence that all of the established human tumor viruses induce hallmarks of autophagy in their host cells. Whether this represents genuine induction of autophagy has not been formally demonstrated in all cases. Only very few tumor virus proteins have been demonstrated to interact directly with autophagy-regulating proteins, and many studies reporting interference with upstream signaling pathways capable of modulating autophagy did not explicitly examine potential effects on autophagy. At least for some of the human tumor viruses, additional studies are necessary to further substantiate their connection to autophagy or to autophagy-like processes, to further elucidate the underlying molecular mechanisms and to provide answers to the key questions of how host cell autophagy affects the viral life cycle and how modulation of the autophagy machinery contributes to the development of virus-induced cancers. The current literature does not provide a uniform picture of whether viruses in general are threatened by or benefit from a functional host cell autophagy machinery and whether they have evolved to inhibit autophagy (or at least some steps in that process) or to exploit it to their own advantage. The answers to these questions may differ for different human tumor viruses.
